# Tumour budding and tumour–stroma ratio in hepatocellular carcinoma

**DOI:** 10.1038/s41416-020-0847-1

**Published:** 2020-05-04

**Authors:** Valtteri Kairaluoma, Niko Kemi, Vesa-Matti Pohjanen, Juha Saarnio, Olli Helminen

**Affiliations:** 1 Cancer and Translational Medicine Research Unit, Medical Research Center Oulu, University of Oulu and Oulu University Hospital, Oulu, Finland; 2 Department of Surgery, Central Finland Central Hospital, Jyväskylä, Finland

**Keywords:** Hepatocellular carcinoma, Hepatocellular carcinoma

## Abstract

**Background:**

Tumour budding and low tumour–stroma ratio (TSR) are associated with poor prognosis in some cancers, but their value in Western hepatocellular carcinoma is unclear. The prognostic value of tumour budding and TSR in hepatocellular carcinoma was examined.

**Methods:**

Some 259 hepatocellular carcinoma patients treated in Oulu University Hospital 1983–2018 were included in this retrospective cohort study. Tumour budding and TSR were analysed from the haematoxylin- and eosin-stained original diagnostic slides, by dividing patients into bud-negative (0 bud) or bud-positive (≥1 bud) groups, and into high TSR (<50%) and low TSR (≥50%) groups. Surgically treated patients (*n* = 47) and other treatments (*n* = 212) were analysed separately. Primary outcomes were overall, and disease-specific 5-year mortality was adjusted for confounding factors.

**Results:**

Surgically treated patients with positive tumour budding had increased 5-year overall (adjusted HR 3.87, 95% CI 1.10–13.61) and disease-specific (adjusted HR 6.17, 95% CI 1.19–31.90) mortality compared with bud-negative patients. In surgically treated patients, TSR had no effect on 5-year overall (adjusted HR 2.03, 95% CI 0.57–7.21) or disease-specific (adjusted HR 3.23, 95% CI 0.78–13.37) mortality. No difference in survival related to tumour budding and TSR in non-surgically treated patients was observed.

**Conclusions:**

Tumour budding is a prognostic factor in surgically treated hepatocellular carcinoma.

## Background

Hepatocellular carcinoma (HCC) is one of the leading causes of cancer mortality, with patients often presenting at an inoperable stage.^1–3^ The known prognostic factors in HCC include clinical and imaging features, serum AFP, tumour size and number, invasion and comorbidity.^3^ Morphological features include tumour grade, subtype, stage, cirrhosis, immunohistochemical expression of CK19, vascular invasion and intrahepatic metastasis.^3^ Tumour budding is an acknowledged prognostic factor in colorectal cancer, and is associated with poor prognosis in several cancer types.^4–13^ Tumour budding is defined as a single tumour cell or a cell cluster of up to four tumour cells at the invasive front of carcinomas, and has been postulated to represent an epithelial-to-mesenchymal transition.^9,14^ For risk stratification of tumour bud counts, two- and three-tier systems have been used.^9^ The prognostic value of tumour budding has been observed in one study in hepatocellular carcinoma.^15^

Tumoural microenvironment plays an important role in tumour progression, growth and metastasis.^16–19^ As a major part of the tumour microenvironment, the stromal component is crucial for tumour development and support.^19^ Tumour–stroma ratio (TSR) is defined as the percentage of tumour cell component relative to the surrounding stroma.^19^ Low TSR (high proportion of stroma) in tumour tissue has been recognised as an important factor of tumour prognosis in various cancer types.^20–25^ The significance of TSR is poorly understood in HCC, especially in the Western population. One study has suggested that low TSR indicates poorer prognosis in HCC.^26^

The aim of this study was to examine the prognostic value of tumour budding in Western HCC patients for the first time, and to evaluate the association between TSR and prognosis in HCC in the population of Northern Finland.

## Methods

### Study design

This study was a retrospective cohort study in a single-institution tertiary care hospital in Northern Finland. A total of 273 patients with histologically confirmed HCC were treated in Oulu University Hospital between January 1983 and March 12, 2018. Of these, representative samples were available from 259 patients for analysis, and were included in the present study. Patient survival data were acquired from Statistics Finland.

### Data collection

The patients were originally identified from the archives using ICD-10 code C22.0& that indicates hepatocellular carcinoma. Diagnoses for each patient were confirmed with histological examination. Clinical data was collected from Oulu University Hospital patient records. Diagnostic haematoxylin- and eosin-stained (HE) histological samples were retrieved from the pathology archives. The 8th edition of TNM classification was used in staging.

### Evaluation of histological samples

The HE-stained slides originally used for diagnostic purposes were used in the present study. Histopathologist (V.-M.P) re-evaluated and confirmed the diagnoses of all included patients. All cases were also re-graded^3^ by histopathologist (V.-M.P.). At first, multiple HE-stained glass slides were viewed with light microscope, and the samples containing both tumour and stroma were selected for further investigation. The histological material consisted of surgical resection samples and core-needle biopsies. If a patient had a surgical resection sample and a biopsy sample available, the surgical resection sample was used for the analysis. Sections were scanned and digitised using Aperio AT2 (Leica Biosystems, Wetzlar, Germany). Tumour budding and tumour–stroma ratio were analysed from scanned sections using Aperio ImageScope by two independent investigators (V.K. and N.K.) blinded to the clinical and outcome data. If the sample estimates were on the different sides of the cut-offs, the sample was re-assessed, and consensus was reached. A few dubious cases were re-assessed by a third investigator (V.-M.P.).

**Fig. 1:**
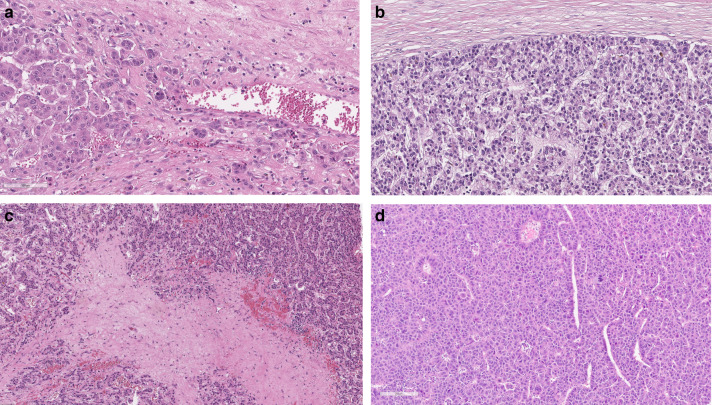
Haematoxylin- and eosin-stained slides of resected hepatocellular carcinomas. The photomicrographs show examples of positive tumour budding (**a**), negative tumor budding (**b**), low TSR (**c**) and high TSR (**d**).

When investigating tumour budding, a bud was defined as a single tumour cell or a cell cluster of up to four tumour cells that seemed to be detached from the main tumour. The hotspot method was used, which is highly recommended when investigating tumour budding in colorectal cancer.^9^ When investigating samples, the tumour area was first screened with low magnification to find the area with most tumour budding. Then, the number of buds were counted from a single field of view at ×200 total magnification (Fig. 1). After assessment, the patients were divided into low- and high-budding groups. The cases were classified as bud-negative if no tumour buds were found and as bud-positive if at least one tumour bud was present, because the median number of tumour buds was found to be zero.

TSR was examined in the same samples as those used for the assessment of tumour budding. Different methods were used for assessing TSR from surgical resection and biopsy samples. The surgical resection samples were viewed at low magnification, and the area with most stroma compared with tumour cells was identified. The area of stroma compared with the area of tumour cells was estimated from a single field of view using ×100 total magnification. The presence of tumour cells was confirmed on all four sides of the field of view before the assessment was performed. The percentage of stroma on a selected area compared with the tumoural cell component was estimated and scored at 10% intervals (10, 20 and 30%) (Fig. 1). Necrosis and normal hepatocytes were excluded. In biopsy samples, the area between the remotest tumour cells of the section was analysed. If the biopsy sample had been shattered into more than two pieces, all of which containing stroma and tumour cells at both ends of the sample, the whole biopsy sample was used for estimation. The amount of stroma on the selected area was scored in the same way as in the surgical resection samples. The cut-off value was set at 50%, and patients were divided into high TSR (<50%) and low TSR (≥50%) groups.

### Outcomes

Primary outcomes of the study were 5-year overall- and disease-specific survival in surgically treated patients. This was defined as death from any cause (overall survival) or HCC (disease-specific survival) during the interval between the date of surgery and death of the patient over 5 years or at the end of the 5-year follow-up.

In non-surgical patients, the primary outcome was 3-year overall- and disease-specific survival due to low expected survival rates.

### Statistical analysis

Mann–Whitney *U* test was used to compare differences between two independent groups with continuous variables. Cohen’s kappa was calculated to analyse interobserver agreement where values between 0.01 and 0.20 indicate none to slight, 0.21–0.40 fair, 0.41–0.60 moderate, 0.61–0.80 substantial and 0.81–1.00 almost perfect agreement.^27^ For categorical data analysis *χ*^2^ test was used. The threshold for significance was set at *P* < 0.05. In all continuous variables, median and interquartile range are presented. Kaplan–Meier method was used to compare survival between groups, and log-rank test was used to analyse statistical differences between groups. Cox regression analysis was used to perform multivariable analysis between groups with the following covariates: sex (female/male), age (continuous), comorbidities (Charlson comorbidity index 0–1, 2 or higher), cirrhosis (no/yes), Child–Pugh points (A, B or C), year of operation/diagnosis (1983–2005 and 2006–2018), tumour grade (1–2 and 3) and stage (1, 2 or higher). Statistical analysis was performed with IBM SPSS statistics 24.0 (IBM Corp., Armonk, NY).

## Results

### Patients

In 259 patients, median age was 72 years (range: 7–93 years, interquartile range 65–79) with male dominance (70.3%). Median follow-up time was 9.1 months (range: 0.1–300.7, interquartile range 2.4–25.8). Of 259 patients, 47 (18.1%) were treated with surgical resection, 24 (9.3%) underwent radiofrequency ablation (RF), laser ablation or percutaneous ethanol injections (PEI), 45 (17.4%) underwent transarterial chemoembolisation (TACE) and 143 (55.2%) patients were given palliative treatment or best supportive care. Four patients treated surgically had only biopsy samples available. Eight non-surgically treated patients had surgical resection samples available. Patients were divided into two groups based on treatment (surgically and non-surgically treated). Median age in patients undergoing surgery was 65 years and in patients treated non-surgically 73 years. Median tumour size in surgically treated patients was 50.0 mm and in non-surgically treated patients 70.0 mm. Differences between surgically and non-surgically treated patients were analysed separately. Patient characteristics are presented in Table 1.

**Table 1:** Baseline characteristics of patients treated surgically or non-surgically.

	Surgery (*N* = 47)	Non-surgically treated patients (*N* = 212)
Age, median (IQR)	65.3 (59.6–71.0)	73.1 (66.6–79.9)
BMI kg/m^2^ (median, IQR)	26.0 (22.9–28.4)	27.2 (24.2–30.4)
Male, *n* (%)	27 (57.4%)	155 (73.1%)
Treatment		
Surgery	47 (100.0%)	–
Local ablation (RF, laser)	–	20 (9.4%)
PEI	–	4 (1.9%)
TACE	–	45 (21.2%)
Palliative/best supportive treatment	–	143 (67.5%)
Sample type		
Surgical resection sample	43 (91.5%)	8 (3.8%)
Core-needle biopsy	4 (8.5%)	204 (96.2%)
Bud-negative	23 (48.9%)	131 (61.8%)
Bud-positive	24 (51.1%)	81 (38.2%)
High TSR	34 (72.3%)	172 (81.1%)
Low TSR	13 (27.7%)	40 (18.9%)
Alcohol consumption		
History of alcohol consumption	5 (10.6%)	72 (34.0%)
No/missing	42 (89.4%)	140 (66.0%)
Liver cirrhosis	15 (31.9%)	79 (37.3%)
Charlson Comorbidity Index		
0	12 (25.5%)	21 (9.9%)
1	19 (40.4%)	59 (27.9%)
2	12 (25.5%)	64 (30.4%)
3	4 (8.5%)	41 (19.3%)
4 or higher	–	26 (12.3%)
Child–Pugh classification		
Child–Pugh A	29 (61.7%)	125 (59.0%)
Child–Pugh B	1 (2.1%)	39 (18.4%)
Child–Pugh C	–	10 (4.7%)
Missing	17 (36.2%)	38 (17.9%)
ASA status, *n* (%)		
Grade I	9 (19.1%)	4 (1.9%)
Grade II	9 (19.1%)	36 (17.0%)
Grade III	29 (61.7%)	146 (68.9%)
Grade IV or higher	–	26 (12.3%)
WHO performance status		
Grade 1	30 (63.8%)	40 (18.9%)
Grade 2	12 (25.5%)	81 (38.2%)
Grade 3	5 (19.6%)	70 (33.0%)
Grade 4 or higher	–	21 (9.9%)
AFP, median (IQR)	7.0 (3.0–260.5)	10 (4.0–157.5)

### Evaluation of tumour budding

For analysis of tumour budding, patients were divided into bud-negative (*N* = 154 (59.5%)) and bud-positive (*N* = 105 (40.5%)) groups (range 0–23 buds). In the bud-negative group, 25 (16.2%) patients, and in the bud-positive, 26 (24.8%) had surgical resection samples available. Cohen’s Kappa value for surgical resection samples was 0.801. In the bud-negative group, 129 (83.8%) patients, and in the bud-positive group 79 (75.2%) had biopsy samples available. Cohen’s Kappa value for biopsy samples was 0.729.

#### Tumour budding in the surgical treatment group

Twenty-three patients (48.9%) were bud-negative and 24 (51.1%) bud-positive (range 0–23 buds). In both groups, the majority of the patients had Child–Pugh classification A (29, 61.8%) and tumour stage I. Vascular invasion was observed in 6 (28.6%) patients in the bud-negative group and in 8 (36.4%) patients in the bud-positive group (*P* = 0.586). No significant differences were observed between the groups. Baseline characteristics of tumour budding in patients treated with surgery are presented in Tables 1 and 2.

**Table 2:** Baseline characteristics of patients treated surgically.

	Bud-negative (*N* = 23)	Bud-positive (*N* = 24)	High TSR (<50%) (N = 34)	Low TSR (≥50%) (*N* = 13)
Major resection	7 (30.4%)	12 (50.0%)	12 (35.3%)	7 (53.8%)
Minor resection	16 (69.6%)	12 (50.0%)	22 (64.7%)	6 (46.2%)
Resection margin				
R0	19 (82.6%)	16 (66.7%)	27 (79.4%)	8 (61.5%)
R1	1 (4.3%)	2 (8.3%)	0 (0.0%)	3 (23.1%)
R2	1 (4.3%)	1 (4.2%)	2 (5.9%)	0 (0.0%)
Postoperative chemo- or radiotherapy	8 (34.8%)	11 (45.8%)	14 (41.2%)	5 (38.5%)
ASG				
No complication	7 (30.4%)	8 (33.3%)	10 (29.4%)	5 (38.5%)
Minor complication	8 (34.8%)	11 (45.8%)	12 (35.3%)	7 (53.8%)
Major complication	8 (34.8%)	5 (20.8%)	12 (35.3%)	1 (7.7%)
Stage				
Stage I	13 (56.5%)	14 (58.3%)	19 (55.9%)	8 (61.5%)
Stage II	9 (39.1%)	7 (29.2%)	11 (32.4%)	5 (38.5%)
Stage III	1 (4.3%)	2 (8.3%)	3 (8.8%)	0 (0.0%)
Stage IV	0 (0.0%)	1 (4.2%)	1 (2.9%)	0 (0.0%)
Vascular invasion				
Yes	6 (28.6%)	8 (36.4%)	10 (31.3%)	4 (36.4%)
No	15 (71.4%)	14 (63.6%)	22 (68.8%)	7 (63.6%)
Tumour localisation				
Right lobe	13 (56.5%)	15 (62.5%)	21 (61.8%)	7 (53.8%)
Left lobe	6 (26.1%)	7 (29.2%)	8 (23.5%)	5 (38.5%)
Both lobes	4 (17.4%)	2 (8.3%)	5 (14.7%)	1 (7.7%)
Unifocal tumour	19 (82.6%)	20 (83.3%)	28 (79.4%)	12 (92.3%)
Tumour grade				
Grade I	3 (13.0%)	1 (4.2%)	2 (5.9%)	2 (15.4%)
Grade II	14 (60.9%)	15 (62.5%)	19 (55.9%)	10 (76.9%)
Grade III	6 (25.1%)	8 (33.3%)	13 (38.2%)	1 (7.7%)
Tumour size (mm), median (IQR)	50.0 (35.0–100.0)	50.0 (31.5–107.5)	60.0 (32.5–100.0)	50.0 (40.5–62.5)

*ASG* Accordion Severity Grading.

No significant differences were observed between the groups.

#### Tumour budding in the non-surgical treatment group

One hundred and thirty-one (61.8%) patients were bud-negative and 81 (38.2%) were bud-positive (range 0–13 buds). Most of the patients in this group had Child–Pugh classification A (125, 59.0%) and tumour stage I. No significant differences were observed between the groups. Baseline characteristics of non-surgically treated patients are presented in Tables 1 and 3.

**Table 3:** Baseline characteristics of patients treated with local ablation, transarterial treatment or palliative treatment.

	Bud-negative (*N* = 131)	Bud-positive (*N* = 81)	High TSR (<50%) (*N* = 172)	Low TSR (≥50%) (*N* = 40)
Postoperative chemo- or radiotherapy	30 (22.9%)	17 (21.0%)	37 (21.5%)	10 (25.0%)
Stage				
Stage I	48 (36.6%)	36 (44.0%)	68 (39.5%)	16 (40.0%)
Stage II	21 (16.0%)	10 (12.3%)	27 (15.7%)	4 (10.0%)
Stage III	36 (27.5%)	22 (27.2%)	48 (27.9%)	10 (25.0%)
Stage IV	24 (18.3%)	12 (14.8%)	28 (16.3%)	8 (20.0%)
Tumour localisation				
Right lobe	65 (49.6%)	31 (38.3%)	79 (45.9%)	17 (42.5%)
Left lobe	14 (10.7%)	16 (19.8%)	25 (14.5%)	5 (12.5%)
Both lobes	51 (38.9%)	34 (42.0%)	67 (39.0%)	18 (45.0%)
Unifocal tumour	59 (45.0%)	37 (45.7%)	77 (44.8%)	19 (47.5%)
Tumour grade				
Grade I	39 (30.5%)	25 (30.9%)	51 (30.2%)	13 (32.5%)
Grade II	74 (57.8%)	45 (55.6%)	98 (58.0%)	21 (52.5%)
Grade III	15 (11.7%)	11 (13.6%)	20 (11.8%)	6 (15.0%)
Tumour size (mm), median (IQR)	75.0 (40.0–110.0)	65.0 (40.0–100.0)	70.0 (40.6–11.0)	72.5 (36.3–100.0)

No significant differences were observed between the groups.

### Evaluation of TSR

Patients were divided into two groups, High TSR (<50%) (*N* = 206, 79.5%) and Low TSR (≥50%) (*N* = 53, 20.5%). Patients with low TSR were more likely to be men than were patients with high TSR (*P* = 0.023). No other differences in patient characteristics between the groups were observed. Surgical resection samples were available for 40 (19.4%) patients with high TSR and for 11 (20.8%) patients with low TSR. Cohen’s Kappa value was 0.876 for surgical resection samples. In the biopsy samples, Cohen’s Kappa value was 0.814.

#### TSR in the surgical treatment group

Of patients treated surgically, 34 (72.3%) had high TSR and 13 (27.7%) low TSR. In the high TSR group, 55.9% had tumour stage I and in the low TSR group 61.5%. Vascular invasion was observed in 10 (31.3%) patients with high TSR and in 4 (36.4%) patients with low TSR group (*P* = 0.755). No significant differences were observed between the groups. Baseline characteristics of TSR in patients treated with surgery are presented in Tables 1 and 2.

#### TSR in the non-surgical treatment group

Of patients treated non-surgically, 172 (81.1%) had high TSR and 40 (18.9%) patients had low TSR. No significant differences were observed between the groups. Baseline characteristics of TSR in non-surgically treated patients are presented in Tables 1 and 3.

### Outcomes of patients

#### Outcomes of patients treated with surgery

In the surgically treated group, 1-, 3- and 5 years, the respective overall survival rates were 86.9, 58.4 and 50.7%. Disease-specific survival rates were 86.9, 65.5 and 59.8%, respectively. Median follow-up time was 41.1 months (range: 1.1–300.7, interquartile range 15.3–89.3).

#### Outcomes of non-surgically treated patients

The respective overall survival rates at 1, 3 and 5 years in the local ablation group were 85.4, 45.6, and 19.9, in the transarterial group 53.9, 28.8 and 7.2% and in the palliative group 27.3, 5.1 and 0.0%. In the local ablation group, disease-specific survival rates at 1, 3 and 5 years were 89.7, 74.1 and 40.5%, in the transarterial group 59.8, 48.5 and 16.2% and in the palliative group 35.8, 8.3,and 0.0%, respectively. Median follow-up time in non-surgically treated patients was 6.9 months (range: 0.1–87.6, interquartile range 1.9–17.7).

### Five-year survival of surgically treated patients

#### Tumour budding, 5-year survival

**Fig. 2:**
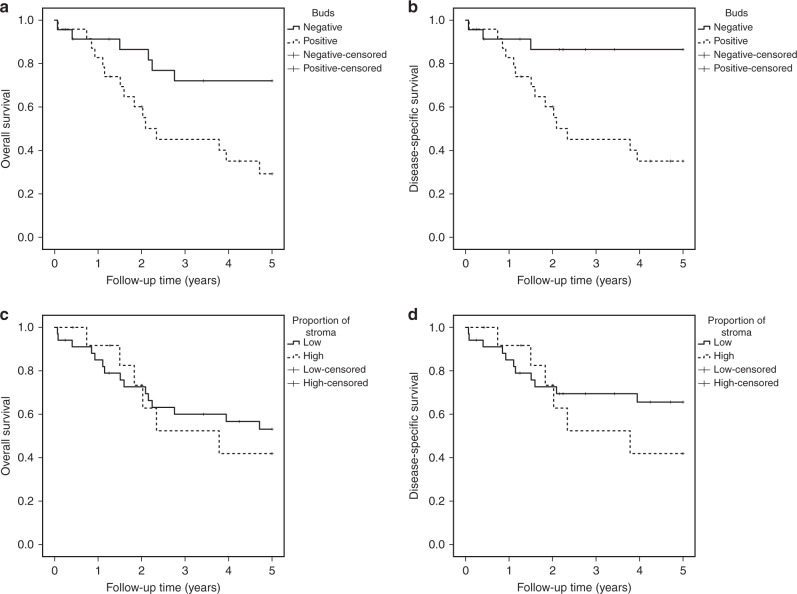
Kaplan-Meier survival curves of hepatocellular carcinoma patients. **a** Overall survival of patients treated with surgery stratified by tumour budding. **b** Disease-specific survival of patients treated with surgery stratified by tumour budding. **c** Overall survival of patients treated with surgery stratified by TSR. **d** Disease-specific survival of patients treated with surgery stratified by TSR.

Overall 5-year survival in bud-negative patients was 72.1% and in bud-positive patients 29.2%, *p* = 0.009 (Fig. 2a). Disease-specific 5-year survival was 86.5% and 35.1%, *p* = 0.002 (Fig. 2b), respectively. In the multivariable analysis adjusted for confounding factors, positive tumour budding was associated with increased risk for 5-year overall (HR 3.87, 95% CI 1.10–13.61) and disease-specific (HR 6.17, 95% CI 1.19–31.90) mortality compared with negative tumour budding (Table 4). In the univariable analysis, positive tumour budding was also associated with increased risk for 5-year overall (HR 3.30, 95% CI 1.27–8.57) and disease-specific (HR 5.64, 95% CI 1.62–19.73) long-term mortality compared with negative tumour budding (Table 4).

**Table 4:** Hazard ratios (HR) with 95% confidence intervals (CI) of mortality comparing patients with hepatocellular carcinoma undergoing surgical resection in Oulu University Hospital 1983–2018.

	Negative budding (*n* = 23) HR (95% CI)	Positive budding (*n* = 24) HR (95% CI)	*P* value	High TSR (*n* = 34) HR (95% CI)	Low TSR HR (95% CI)	*P* value
*5-year overall mortality*						
Crude	1 (reference)	3.30 (1.27–8.57)	0.014	1 (reference)	1.20 (0.46–3.10)	0.711
Adjusted^a^	1 (reference)	3.87 (1.10–13.61)	0.035	1 (reference)	2.03 (0.57–7.21)	0.276
*5-year disease-specific mortality*						
Crude	1 (reference)	5.64 (1.62–19.73)	0.007	1 (reference)	1.55 (0.57–4.21)	0.388
Adjusted^a^	1 (reference)	6.17 (1.19–31.90)	0.030	1 (reference)	3.23 (0.78–13.37)	0.105

Follow-up ended on December 31, 2017. In patients operated in 2018, follow-up ended 30 days after surgery.

^a^Adjustment for age (continuous), sex (female/male), Charlson Comorbidity Index (0–1, 2 or higher), stage (1, 2 or higher), cirrhosis (no/yes), year of surgery/diagnosis (1983–2005 and 2006–2018), Child–Pugh index (A, B or C) and tumour grade (1–2 and 3).

#### Tumour–stroma ratio, 5-year survival

Overall 5-year survival in patients with high TSR was 53.1% and in patients with low TSR 41.9%, *p* = 0.711 (Fig. 2c). Disease-specific 5-year survival was 65.6% and 41.9%, *p* = 0.384 (Fig. 2d), respectively. In the multivariable analysis adjusted for confounding factors, low TSR was not associated with increased risk for 5-year overall mortality (HR 2.03, 95% CI 0.57–7.21) or disease-specific mortality (HR 3.23, 95% CI 0.78–13.37) compared with high TSR (Table 4). In the univariable analysis, no differences between groups were observed (Table 4).

### Three-year survival of non-surgically treated patients

Non-adjusted long-term survival was not compared in non-surgically treated patients due to heterogeneity in treatments. In the adjusted model, there was no significant difference in non-surgically treated patients in tumour budding and TSR (Table 5).

**Table 5:** Hazard ratios (HR) with 95% confidence intervals (CI) of mortality comparing patients with hepatocellular carcinoma undergoing non-surgical treatment in Oulu University Hospital 1983–2018.

	Negative budding (*n* = 131) HR (95% CI)	Positive budding (*n* = 81) HR (95% CI)	P value	High TSR (*n* = 172) HR (95% CI)	Low TSR (*n* = 40) HR (95% CI)	*P* value
*3-year overall mortality*						
Crude^a^	1 (reference)	0.70 (0.51–0.97)	0.031	1 (reference)	1.06 (0.72–1.57)	0.756
Adjusted^b^	1 (reference)	0.81 (0.58–1.15)	0.241	1 (reference)	1.01 (0.66–1.55)	0.965
*3-year disease-specific mortality*						
Crude^a^	1 (reference)	0.64 (0.44–0.93)	0.020	1 (reference)	0.97 (0.61–1.52)	0.884
Adjusted^b^	1 (reference)	0.74 (0.50–1.11)	0.143	1 (reference)	0.89 (0.54–1.47)	0.643

Follow-up ended on December 31, 2017. In patients operated in 2018, follow-up ended 30 days after surgery.

^a^Crude was adjusted with treatment (local ablation/TACE/palliative).

^b^Adjustment for age (continuous), sex (female/male), Charlson Comorbidity Index (0–1, 2 or more), stage (1, 2 or more), cirrhosis (no/yes), year of surgery/diagnosis (1983–2005 and 2006–2018), Child–Pugh index (A, B or C) and tumour grade (1–2 and 3) (local ablation/TACE/palliative).

## Discussion

The results of this study suggest that assessment of tumour budding is reproducible and an independent prognostic factor in hepatocellular carcinoma patients treated surgically.

Some strengths and limitations in this study must be noted. The strengths of this study are homogeneous study population and single geographical area where the diagnosis and treatment occurred in the same hospital without selection bias. All patients were treated in a single centre with full access to patient records. A single-institution study, however, causes some limitations due to the relatively small number of patients and wide confidence intervals, especially in the surgical cohort. Also, the long time period of 35 years (1983–2018) may cause confounding due to the improvements in HCC treatment over the years. Nevertheless, this limitation was taken into account by adjusting the multivariate analysis for year of surgery and other confounders. In recent cases, 5-year follow-up was not reached, which could cause confounding, although follow-up time was taken into account in statistical analyses. Furthermore, with the present sample size, we were restricted to a negative/positive cut-off in tumour budding instead of searching for the optimal cut-off value. According to the regression analysis, mortality risk increases with positive tumour budding, and future studies will need to set an optimal cut-off. Good interobserver repeatability was observed in both surgical resection and biopsy samples. The evaluation technique used in core biopsy samples was used for the first time. Even though the Cohen’s Kappa value was good, no significant differences were observed in the study groups with only biopsy samples available. This could result from underestimation of tumour budding and TSR from biopsy samples, which needs to be confirmed in future studies. Difference in samples was also the reason why survival in the surgical and non-surgical cohorts was not compared. In non-surgical patients, tumour budding and TSR were not prognostic, possibly due to advanced tumour stage at diagnosis and short survival as shown in stage IV colorectal cancer.^28^

The present study is the first to analyse tumour budding in Western hepatocellular carcinoma cohort. In the Chinese study of 423 HCC cases mainly related to hepatitis B virus infection, tumour budding was an adverse prognostic factor,^15^ suggesting that budding is clinically important, regardless of HCC aetiology. Tumour budding has previously been studied in a variety of carcinomas.^4,8,11–13,29,30^ A systematic review for colorectal cancer showed that exhibiting tumour budding was associated with lymph node positivity, higher risk for recurrence and higher risk of cancer-related death at 5 years.^30^ The studies included varied in cut-off for the presence or absence of tumour budding up to >9 buds. A Finnish study evaluating gastric cancer reported a similar finding that 5-year survival was lower in the high-budding group.^29^ The cut-off in this study was set at 10 buds.^29^ A Chinese study showed that high tumour budding (8 or more buds) was associated with the presence of lymphovascular invasion, larger tumour size and poorer clinical outcome compared with a low tumour budding group in breast cancer.^8^ Also, similar results have been reported in pancreatic cancer,^11^ oesophageal squamous cell carcinoma^13^ and in head and neck squamous cell carcinoma.^4^

One earlier study of TSR in hepatocellular carcinoma has been published.^26^ In a Chinese study, TSR was an independent prognostic factor for HCC patients after liver resection or transplantation.^26^ The same 50% cut-off was set as in our study, and 0.870 kappa value was achieved, which was similar to that in our study. Low TSR patients’ overall survival rates were significantly lower than those of high TSR patients, and this finding was repeated after adjusting for confounding factors.^26^ We could not repeat this finding. The difference in point-estimate magnitude and statistical significance between the studies may be due to our small sample size of surgically treated patients, or potentially by differences in aetiology, namely alcohol in Finland^31,32^ and viral infections in China.^33^

Previously, TSR has been studied in a variety of cancers.^18,19,21–24,26,34,35^ In oesophageal adenocarcinoma, patients with low TSR had poorer disease-free and overall survival.^18^ In an Italian study, a low TSR was identified as an independent marker for poor prognosis in patients with gastric cancer.^21^ Similar results have been observed in breast^22^ and cervical cancer.^35^ There are also studies reporting a negative association between TSR and survival, for example in oesophageal cancer.^36^

Tumour budding is widely believed to provide an important histological basis for invasion and metastasis.^37^ The findings of downregulation of epithelial markers and upregulation of mesenchymal markers have implied that tumour budding is the morphological expression of epithelial-to-mesenchymal transition. However, it has also been observed that most epithelial-to-mesenchymal transition processes in tumour buds are not complete, giving rise to the notion that tumour buds undergo partial epithelial-to-mesenchymal transition.^37^

The mechanism leading to poorer prognosis of patients with low TSR is not yet fully understood. Complex interactions between stromal and cancer cells have been suggested to be a part of cancer development.^20^ Cancer-associated fibroblasts may play an important role during tumour development from preneoplastic to metastatic state.^38^ A number of studies have implicated reactive stroma, including activated fibroblasts, in accelerating carcinoma development.^38^ The complex tumour-related stroma components, including the extracellular matrix and various cell types, are known to assist the communication between stromal and cancer cells.^19^ All in all, tumour budding and TSR are under intense research in order to learn more about tumour development and interaction between tumour and stromal cell components.

The results of the present study have clinical and research-related implications. Our study showed for the first time that tumour budding is an independent prognostic factor in Western patients with hepatocellular carcinoma treated surgically. The analysis of tumour budding can be reliably replicated and routinely analysed from HE-stained slides without additional immunohistochemistry or costs. According to our study, tumour budding can be used in daily clinical practice, but validation studies are still needed. Also, more studies are needed to confirm the prognostic value of TSR in hepatocellular carcinoma.

In conclusion, the results of this study show that tumour budding is an independent prognostic factor in hepatocellular carcinoma.

## Data Availability

Anonymised data are available from the corresponding author upon request. Sharing the data will require additional ethical approval.
